# Expression profiles of *cIRF6, cLHX6 *and *cLHX7 *in the facial primordia suggest specific roles during primary palatogenesis

**DOI:** 10.1186/1471-213X-6-18

**Published:** 2006-03-24

**Authors:** Belinda J Washbourne, Timothy C Cox

**Affiliations:** 1 School of Molecular and Biomedical Science, University of Adelaide, South Australia, Australia; 2 Australian Craniofacial Institute, North Adelaide, South Australia, Australia; 3 Department of Anatomy and Cell Biology, Monash University, Victoria, Australia

## Abstract

**Background:**

The LIM-homeodomain transcription factors LHX7 and LHX6 have been implicated in palatogenesis in mice and thus may also contribute to the incidence of isolated palatal clefts and/or clefts of the lip and primary palate (CL/P) in humans. Causative mutations in the transcription factor IRF6 have also been identified in two allelic CL/P syndromes and common polymorphisms in the same gene are significantly associated with non-syndromal CL/P in different populations.

**Results:**

Here we report the isolation of chick orthologues of *LHX7*, *LHX6 *and *IRF6 *and the first characterisation of their profiles of expression during morphogenesis of the midface with emphasis on the period around formation of the primary palate. *LHX7 *and *LHX6 *expression was restricted to the ectomesenchyme immediately underlying the ectoderm of the maxillary and mandibular primordia as well as to the lateral globular projections of the medial nasal process, again underlying the pre-fusion primary palatal epithelia. In contrast, *IRF6 *expression was restricted to surface epithelia, with elevated levels around the frontonasal process, the maxillary primordia, and the nasal pits. Elsewhere, high expression was also evident in the egg tooth primordium and in the apical ectodermal ridge of the developing limbs.

**Conclusion:**

The restricted expression of both *LHX *genes and *IRF6 *in the facial primordia suggests roles for these gene products in promoting directed outgrowth and fusion of the primary palate. The manipulability, minimal cost and susceptibility of chicks to CL/P will enable more detailed investigations into how perturbations of *IRF6, LHX6 *and *LHX7 *contribute to common orofacial clefts.

## Background

Many genes have been implicated in syndromal and/or non-syndromal cleft lip with or without palate (CL/P). The majority of these candidate genes show expression in the facial ectoderm, including *MSX1*, *BMP4*, *PVRL1*, *MID1 *and *p63*, although some (eg. *MSX1 *and *BMP4*) also have key roles in the mesenchyme [1]. In addition to coordinating mesenchymal outgrowth, the facial ectoderm also plays a number of other pivotal roles in facial morphogenesis, including facilitating the initial contact of the converging processes and the subsequent elimination of the epithelial seam in a manner that is likely analogous to that which occurs during formation of the more studied secondary palate. Recently, mutations in the *IRF6 *(Interferon Regulatory Factor 6) gene were shown to cause the allelic disorders, Van der Woude and Popliteal pterygium syndromes, both of which have CL/P as a major clinical feature [2,3]. Significantly, common polymorphisms in *IRF6 *have also been found to account for up to 12% of the contribution to the high incidence of non-syndromal CL/P, highlighting it as one of the most significant CL/P loci identified to date [4]. Although the exact physiological function of this gene is not known, preliminary findings in mice found that *Irf6 *is expressed in the medial edge epithelia of the fusing secondary palatal shelves, tooth buds, hair follicles and skin [5]. Surprisingly, however, the expression of *IRF6 *has not been reported during development and closure of the primary palate, an event which is distinct both in terms of embryological timing and underlying genetics from that of the secondary palate.

Several secreted factors emanating from the facial ectoderm, including fibroblast growth factor 8 (FGF8) and bone morphogenic protein 4 (BMP4) induce mesenchymal expression of genes such as *Msx1 *and *Msx2*, that promote mesenchymal cell proliferation and prominence outgrowth [6,7]. Evidence suggests that the mesenchymal LIM-Homeodomain (HD) encoding gene, *Lhx7 *(also referred to as *Lhx8 *[8] and *L3 *[9]), is similarly under the control of ectodermal-derived signals, namely Fgf8 [10-13]. In the mouse, *Lhx7 *and its close homologue, *Lhx6*, were reported to be expressed only in the maxillary and rostral mandibular processes, palatal shelves and basal forebrain [9,10,14]. In *Lhx7*-knockout mice, an isolated secondary palatal cleft was the only reported feature: the secondary palatal shelves formed and elevated normally but failed to properly contact and fuse [15]. Of note however, is that most inbred mouse strains rarely display lateral facial clefts analogous to CL/P in humans. This is probably due in part to the altered growth rates that give rise to the elongated facial morphology although differences in sensitivity to gene dosage or redundancies between related genes may also play a role [1]. Consistent with this, mutations in *MSX1 *in humans are associated with clefts of the primary palate whereas knockout of both *Msx1 *and *Msx2 *are required to produce a primary palate cleft in mice [16]. In contrast, the chick in some ways provides a more suitable model system for studies on primary palatal clefting as this species, like humans, shows greater susceptibility to this anomaly. Here, we have isolated chick cDNAs orthologous to human *IRF6*, *LHX7 *and *LHX6 *and investigated their profile of expression during morphogenesis of the midface with an emphasis on the period around formation of the primary palate.

## Results

### Sequence conservation of LHX7, LHX6 and IRF6 between chick, mouse and human

**Figure 1:**
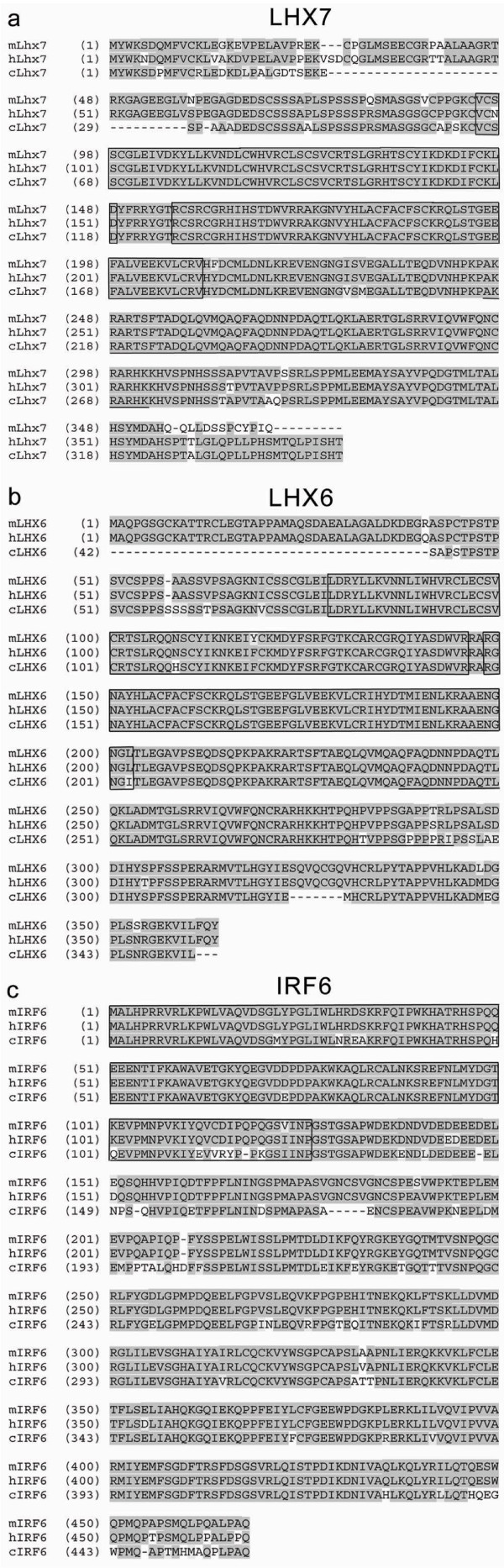
**Protein alignments of mouse, chick and human LHX7 (a), LHX6 (b) and IRF6 (c). **cLHX7 displays 89% and 95% identity with mouse Lhx7 and human LHX7, respectively. cLHX6 displays 94% identity and 99% similarity to both human LHX6 and mouse Lhx6. cIRF6 displays 83% identity, 99% similarity with human IRF6 and mouse Irf6. Legend: The LIM domains of LHX6/7 and DNA-binding domain of IRF6 are boxed. The homeodomain of LHX6/7 is underlined.

*cLHX7*: chEST766i11 was shown to encode a protein with 89% and 95% identity to mouse and human Lhx7/LHX7, respectively (Fig 1a). *cLHX6: *chEST365j8 represented a partial sequence (708 bp) encoding two LIM domains and the 5' end of a HD which displayed 94% identity (99% similarity) to both human and mouse LHX6/Lhx6 (Fig 1b). *cIRF6*: chEST58f7 represented a partial sequence of 1665 bp that encoded the C-terminal two thirds (304 amino acids) with 83% identity (99% similarity) to human and mouse IRF6/Irf6 (Fig 1c).

### Expression of *cLHX6 *and *cLHX7 *in the facial primordia

**Figure 2:**
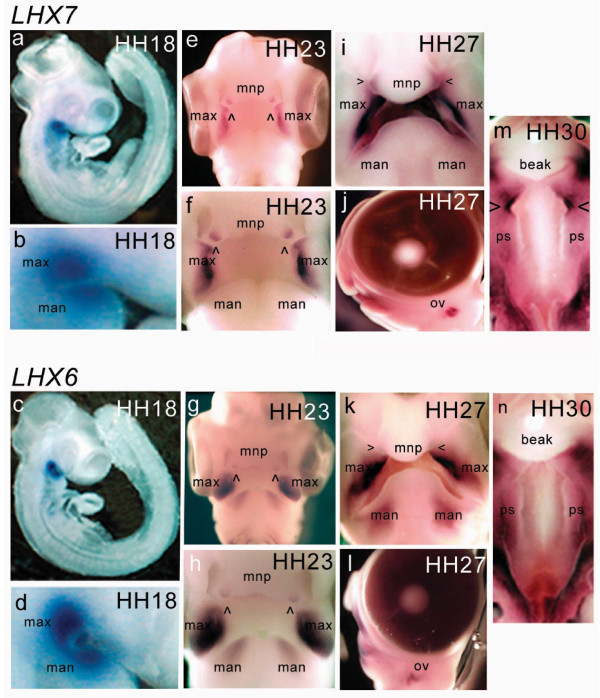
**Expression pattern of *cLHX7 *and *cLHX6 *in the developing chick embryo. ***cLHX7 *(top panel) and *cLHX6 *(bottom panel) were restricted to the ventral extremities of the maxillary primordia and the rostral tip of the mandibular primordia before and after fusion of the maxillary primordia and medial nasal process during formation of the primary palate (a – i, k). From around HH27, *cLHX6 *expression was dispersed throughout the mandibular primordia (k). *cLHX7 *and *cLHX6 *expression was detected in the pre-fusion zone of the medial nasal process, prior to fusion with the maxillary primordia (e, f, g, h). The expression in the medial nasal process remained in the mesenchymal bridge of the beak after fusion (i, k). *cLHX7 *and *cLHX6 *expression was detected in the mesenchyme throughout the palatal shelves at HH30 (m, n). *cLHX7 *specifically displayed increased expression on the anterior tips of the developing shelves (m). Both *cLHX7 *and *cLHX6 *expression was detected in the otic vesicle from HH25 to HH30 (j, l). Abbreviations: *max*: maxillary primordia; *man*: mandibular primordia; *mnp*: medial nasal process; *ov*: otic vesicle; *ps*: palatal shelves.

**Figure 3:**
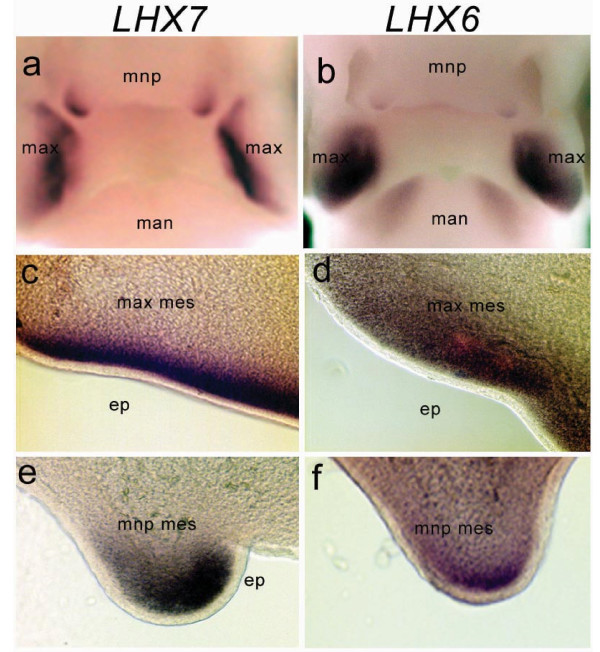
**Vibratome sections of whole-mount *in situ *hybridization embryos**. Sectioning of stage HH23 whole mount *in situ *hybridization embryos indicates that both *LHX7 *(left column) and *LHX6 *(right column) show expression in the neural crest-derived mesenchyme of the first branchial arch (maxillary primordia shown) (c, d) and the lateral globular masses at the edges of the medial nasal process (e, f) restricted to the region directly subjacent to the ectoderm. Abbreviations: *max mes*: maxillary primordia mesenchyme; ep: epithelium; mnp: medial nasal process

*cLHX6 *and *cLHX7 *expression was initially detected by whole-mount *in situ *hybridization at Hamburger-Hamilton stage (HH)15 and remained detectable up to HH30 (Fig 2). Strong expression was found ventrally along the length of the maxillary primordia and the rostral portion of the mandibular primordia. Maxillary expression remained high post-fusion with the medial nasal process (Fig 2a–i, k). In the mandibular primordia, *cLHX6 *expression remained strong whereas *cLHX7 *appeared to gradually diminish from stage HH23. Of note, expression of both *cLHX7 *and *cLHX6 *was also detected in mesenchyme immediately underlying the pre-fusion epithelia of the globular projections of the medial nasal process from HH22 (arrowheads in Fig 2e, f, g, h). *cLHX7 *expression was far more pronounced than *cLHX6 *in this region and remained detectable in the mesenchymal bridge of the beak up to HH30, albeit at a much lower level (not shown). Vibratome sections of these embryos revealed that expression of *cLHX7 *and *cLHX6 *was restricted to ectomesenchyme directly subjacent to the facial ectoderm (Fig 3). This expression profile resembles in part that of the Wnt inhibitor, Dkk1 [17] and is consistent with the evidence indicating *Lhx7 *and *Lhx6 *are under the regulation of signals emanating from the ectoderm [12,18,19]. Expression of both genes was also detected later in the palatal shelf mesenchyme at HH30 (Fig 2m, n), with *cLHX7 *uniquely displaying strong expression on the anterior tips of the developing shelves. Expression of both *cLHX7 *and *cLHX6 *was also detected in the basal forebrain (data not shown) and the otic vesicle from HH25 to HH30 (Fig 2j, l).

### Expression of *cIRF6 *in HH20-29 embryos

**Figure 4:**
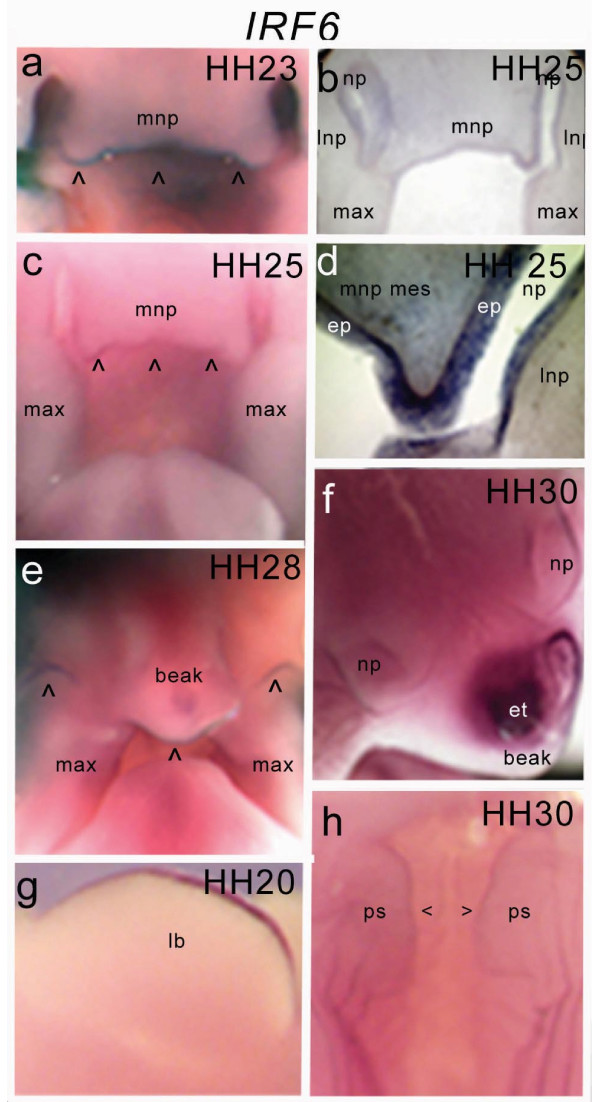
**Expression of *IRF6 *in the developing chick embryo. **Expression is restricted to facial ectoderm. Whole-mount *in situ *hybridisation (a, c, e) and vibratome sections of whole-mount embryos (b, d) revealed notable *IRF6 *expression in the epithelia surrounding the frontonasal process, the maxillary primordia, and the nasal pits. *IRF6 *expression was also detected in the ectoderm of the leading edges of the palatal shelves and in the ridges of the primitive oral cavity at HH30 (h). High *IRF6 *expression was also found in the apical ectodermal ridge of the limb buds (g) and in the egg tooth primordium (f).

*cIRF6 *expression was detected by whole-mount *in- situ *hybridization throughout the ectoderm of the craniofacial structures of HH20-29 embryos (Fig 4). Vibratome sections of whole-mount embryos revealed *IRF6 *levels generally very low but were elevated in the epithelia covering the frontonasal process, the maxillary primordia, and the nasal pits (Fig 4b, d). Expression in the leading edges of the medial nasal process, which ultimately fuse with the maxillary primordia during formation of the primary palate, disappeared with the elimination of epithelia and formation of the mesenchymal bridge. *cIRF6 *expression was also detected in the ectoderm of the leading edges of the developing palatal shelves as well as in the ridges of the primitive oral cavity at HH29 (Fig 4h). High *cIRF6 *expression was also detected in the apical ectodermal ridge of the limb buds (Fig 4g). Notably, expression was also very high in the egg tooth primordium from HH27 (Fig 4f).

## Discussion

Here we have isolated the chick orthologues of *LHX7*, *LHX6 *and *IRF6 *and shown a high degree of sequence conservation with their mouse and human counterparts, suggesting evolutionary conserved functions for these proteins. It should be noted that despite repeated attempts at amplification of mRNA/cDNA across the equivalents of exons 1 to 3 and analysis of available chick genomic sequence, *cLHX7 *did not contain the equivalent of human and mouse exon 2. As this exon does not encode a known functional domain and its absence maintains the reading frame, it is likely that *cLHX7 *also encodes a functional LIM-HD transcription factor. Interestingly, compared to the mouse, chick and human *LHX7 *had an additional 4 bp in the last coding exon producing a C-terminus with an additional nine amino acids. The validity of the mouse *Lhx7 *sequence over this region was confirmed by sequencing murine (Swiss) genomic DNA. That the additional 4 bp is also evident in rat *LHX7 *indicates this 4 bp deletion likely represents a recent evolutionary event that may be restricted to the murine lineage. The biological significance of this must await determination.

In order to determine whether the chick would provide an appropriate model with which to investigate the roles of LHX7, LHX6 and IRF6 in craniofacial development and CL/P, their respective expression patterns around the time of primary palate morphogenesis were determined. Similarly to the mouse, *cLHX7 *and *cLHX6 *were expressed in ectomesenchyme of the maxillary and mandibular primordia [9,10] although in contrast to the mouse, the mandibular expression of *cLHX7 *was not prominent. Differential expression of *cLHX7 *and *cLHX6 *in the mandibular primordia was also evident at the anterior tips of the palatal shelves suggesting thee two genes are under distinct regulatory control which is consistent with results from Cre-mediated Fgf8-knockout mice [11] in which *Lhx7 *but not *Lhx6 *expression was lost.

Of particular interest, our expression studies in the chick have identified unique *LHX7 *and *LHX6 *expression domains. We detected strong *cLHX7 *and *cLHX6 *expression in the mesenchyme immediately underlying the pre-fusion epithelia of the medial nasal process, from around HH22, which remained in the mesenchymal bridge post fusion. This expression has not previously been reported for the mouse and importantly suggests a role for LHX7 and LHX6 in outgrowth/survival of the medial nasal process during formation of the primary palate. *cLHX7 *and *cLHX6 *expression was also detected in the otic vesicle (from HH25 to HH30) a site of expression also not been reported in mice or any other species and therefore may be unique to the chick.

The strong expression in maxillary and medial nasal mesenchyme subjacent to the pre- and post fusion ectoderm indicate that *LHX7 *and *LHX6 *would be good candidate genes for craniofacial anomalies, in particular CL/P despite the isolated secondary palate cleft in *Lhx7 *knock-out mice. In this regard, *hLHX7 *localizes to chromosome 1p31-4 (and not 4q as previously suggested [20]) and is found less than 1.4 Mb from marker D1S1665 which showed the most significant linkage in one cohort of non-syndromal CL/P cases [20]. In fact, this same region produced the only positive lod score for an individual Finnish family presenting with Van der Woude syndrome-like features [21]. These data and the results reported herein put forward a case for screening patients with non-syndromal CL/P or *IRF6 *mutation negative Van der Woude syndrome for mutations in *LHX7*.

*IRF6 *is mutated in Van der Woude and Popliteal pterygium syndromes [2,3] and has recently been identified as one of the most significant non-syndromal CL/P loci to date [4]. This report is the first to describe *IRF6 *expression in the facial primordia prior to and during morphogenesis of the primary palate and supports the notion of a primary ectodermal defect in patients harboring mutations in *IRF6*. In concordance with the later embryonic stages assessed in the mouse [5], *cIRF6 *was similarly detected in the leading edge ectoderm of the palatal shelves and the ridges of the primitive oral cavity in the chick. Interestingly, we also detected some unique expression domains of *IRF6*, which may be specific to the chick. Strong *IRF6 *expression was detected in the egg tooth primordium indicating it as an excellent marker for this structure. Like other genes that are expressed in the ectoderm of the developing face such as *SHH *and *BMP4 *[22,23], high *IRF6 *expression was also detected in the apical ectodermal ridge of the limb buds, which is consistent with the presence of limb hypoplasia or agenesis of digits, syndactyly, as well as valgus or varus deformities of the feet seen in Popliteal pterygium syndrome [24].

## Conclusion

The data presented herein shows both highly conserved and unique temporal and spatial expression of *LHX7*, *LHX6 *and *IRF6 *in the chick, particularly in the facial primordia around the time of their fusion to form the primary palate. The manipulability, minimal cost and susceptibility of chicks to CL/P will enable more detailed investigations into the functions of these genes in midfacial development and their role in contributing to common orofacial clefts.

## Methods

### Isolation of *cLHX7, cLHX6 *and *cIRF6*

Full-length murine *Lhx7, Lhx6 *and *Irf6 *cDNAs (Genbank: AJ000338, AB031040, NM_016851, respectively) were used to BLAST the BBSRC chick expressed sequenced tag (EST) database [25]. Clones that displayed a high degree of homology were purchased from MRC GeneService (Cambridge, UK) then purified and completely sequenced using vector primers. Automated DNA sequencing was performed by cycle sequencing with Applied Biosystems Dye Terminator chemistry v3. cDNA sequences and predicted amino acid analyses and alignments were performed locally using Vector NTI and via the internet using BLAST at NCBI [26]. Primers used to amplify and sequence *mLhx7 *exons 6–9 were as follows: **mLx7ex6-9f**: 5'-TGA-AGA-GAG-AAG-TGG-AGA-ACG-3'; **mLx7ex6-9f: **5'-TGG-GCA-AGA-GGA-TGT-TC-3'.

### Whole-mount in situ hybridization on chick embryos

Fertilized chicken eggs were purchased from HiChick (Gawler, South Australia) and incubated at 36°C, 80% humidity for the appropriate times. Embryos were staged according to Hamburger and Hamilton [27]. Embryos were dissected from the eggs in cold phosphate buffered saline (PBS), fixed in 4% paraformaldehyde (PFA) in PBS either at room temperature for 2 hours or overnight at 4°C and then dehydrated through a series of increasing methanol/PBT (PBS + 0.1% Triton X) washes [28]. For whole-mount *in situ *hybridization, digoxygenin-labeled sense and anti-sense RNA probes were generated by *in vitro *transcription as follows: *cLHX7*: a 1.2 kb *HindIII *fragment from chick EST 766i11 (chEST766i11) was subcloned into appropriately restricted pBluescript and the resultant plasmid linearised using *NotI *(antisense) and *ClaI *(sense) and transcribed using T3 and T7 polymerases, respectively. *cLHX6*: the 709 bp chEST365j8 was linearised using *NotI *(anti-sense) and *HindIII *(sense) and transcribed using T3 and T7 polymerases, respectively. *cIRF6*: chEST58f7 was digested with *SacI *and subcloned into pBluescript. The resultant 889 bp fragment linearised with *MscI *(antisense) and *EcoRI *(sense) and transcribed using T3 and T7 polymerases, respectively. Hybridisation, washes and probe detection were carried out on whole or dissected chick embryos from HH 10–30 according to Xu and Wilkinson [28]. Post-hybridisation, HH23, 27 and 30 chick embryos were fixed in 4% PFA, embedded in 7% low melting agarose (Sigma A2576) and sectioned with a vibratome to a thickness of 100μm. All chick embryo work was reviewed and approved by the University of Adelaide Animal Ethics Committee.

## Authors' contributions

BJW participated in the design of the study, carried out all described experiments and drafted the manuscript. TCC conceived, designed and coordinated the study, advised on protein alignments, and helped draft the manuscript. Both authors read and approved the final manuscript.

## Note added in proof

During review of our article, a study by Inoue *et al *(Inoue M, Kawakami M, Tatsumi K, Manabe T, Makinodan M, Matsuyoshi H, Kirita T, Wanaka A. Expression and regulation of the LIM homeodomain gene L3/Lhx8 suggests a role in upper lip development of the chick embryo. *Anat Embryol*. 2006 Epub ahead) was published that similarly reported restricted mesenchymal expression of chick *LHX7 *and demonstrated that it, like its murine counterpart, appears to be under the regulatory control of epithelial signals including FGF8.
